# Lipid droplets hypertrophy: a crucial determining factor in insulin regulation by adipocytes

**DOI:** 10.1038/srep08816

**Published:** 2015-03-06

**Authors:** Bahram Sanjabi, Monireh Dashty, Behiye Özcan, Vishtaseb Akbarkhanzadeh, Mehran Rahimi, Manlio Vinciguerra, Felix van Rooij, Saad Al-Lahham, Fareeba Sheedfar, Theo G. van Kooten, C. Arnold Spek, Ajda T. Rowshani, Johannes van der Want, Rene Klaassen, Eric Sijbrands, Maikel P. Peppelenbosch, Farhad Rezaee

**Affiliations:** 1 Department of Genetics, University Medical Center Groningen, Groningen. The Netherlands; 2 Department of Gastroenterology and Hepatology, Erasmus Medical Center, University of Rotterdam, Rotterdam, The Netherlands; 3 Department of Cell Biology, University Medical Center Groningen, University of Groningen, Groningen, The Netherlands; 4 Department of endocrinology, Erasmus Medical Center, Rotterdam, The Netherlands; 5 Institute Center-45, Medical University of Amsterdam, University of Amsterdam, The Netherlands; 6 Faculty of Medical Science, University Medical Center Groningen, University of Groningen, Groningen, The Netherlands; 7 Institute for Liver and Digestive Health, Division of Medicine, University College London (UCL), London, UK; 8 Gastroenterology Unit, IRCCS Casa Sollievo della Sofferenza, San Giovanni Rotondo, Italy; 9 Department of cardiovascular genetics, Metabolism, Erasmus Medical Center, Rotterdam, The Netherlands; 10 Department of Medical Biology, University Medical Center Groningen, University of Groningen, Groningen, The Netherlands; 11 Department of Physiology, Radboud University Medical Center, Nijmegen, The Netherlands; 12 Department of Biomedical Engineering, University Medical Center Groningen, University of Groningen, Groningen, The Netherlands; 13 Department for Experimental and Molecular Medicine, Academic Medical Center, University of Amsterdam, The Netherlands; 14 Department of Internal Medicine, section Nephrology and Transplantation, Erasmus Medical Center, Rotterdam, the Netherlands; 15 Department of Laboratory Medicine, Children's and Women's Health, Norwegian University of Science and Technology, Trondheim, Norway; 16 Department of Surgery, Bariatric Surgery, Maas City Hospital, Rotterdam, The Netherlands

## Abstract

Lipid droplets (LDs) hypertrophy in adipocytes is the main cause of energy metabolic system dysfunction, obesity and its afflictions such as T2D. However, the role of adipocytes in linking energy metabolic disorders with insulin regulation is unknown in humans. Human adipocytes constitutively synthesize and secrete insulin, which is biologically functional. Insulin concentrations and release are fat mass- and LDs-dependent respectively. Fat reduction mediated by bariatric surgery repairs obesity-associated T2D. The expression of genes, like *PCSK1* (proinsulin conversion enzyme), *GCG* (Glucagon), *GPLD1*, *CD38* and *NNAT,* involved in insulin regulation/release were differentially expressed in pancreas and adipose tissue (AT). *INS* (insulin) and *GCG* expression reduced in human AT-T2D as compared to AT-control, but remained unchanged in pancreas in either state. Insulin levels (mRNA/protein) were higher in AT derived from prediabetes BB rats with destructed pancreatic β-cells and controls than pancreas derived from the same rats respectively. Insulin expression in 10 human primary cell types including adipocytes and macrophages is an evidence for extrapancreatic insulin-producing cells. The data suggest a crosstalk between AT and pancreas to fine-tune energy metabolic system or may minimize the metabolic damage during diabetes. This study opens new avenues towards T2D therapy with a great impact on public health.

In 1922, Banting and Macleod discovered insulin expression in highly specialized endocrine β-cells within islets of Langerhans in the pancreas^1^. On the one hand, insulin secretion is known to be affected by immunoreactivity to insulin in type 1 diabetes (T1D) in humans. On the other hand, a second defect essential for development from insulin resistance (IR) to type 2 diabetes (T2D) is the inability of endocrinal pancreatic β-cells to produce the required levels of insulin that maintain normal blood glucose levels. It has also been shown that the insulin sensitivity decreases in an obesity state and thereby suppresses β-cells functionality^2^,^3^. In addition, obesity is implicated in multiple pathophysiological complications such as T2D and cardiovascular disease (CVD)^4^,^5^,^6^. Since obesity is determined by the excessive mass of adipose tissue (AT) and in particular adipocytes, adipocytes play an important role in the development of obesity^7^,^8^. Hypertrophy of adipocytes is the main cause of obesity^7^,^8^,^9^. These results from the excessive storage of energy in the form of triglycerides (TGs) in lipid droplets (a monolayer membrane with a structure similar to very low-density lipoprotein^10^) within adipocytes, which links to obesity and to IR. Of note, AT is considered as the largest endocrine organ in humans^7^,^8^,^9^,^11^.

In spite of increasing studies on the properties of AT and in particular adipocytes, the mechanisms that lead to obesity-induced pathophysiological states are still poorly understood. Moreover, the advent of new technology that allows the characterization of entire transcriptomes and proteomes^12^ had also led to the hope that the properties of AT might be revealed to help discover new therapeutic avenues. However, hitherto, the results have not lived up to their expected promises, prompting us to perform a well-designed and in-depth study on human primary subcutaneous and visceral preadipocytes and adipocytes.

Since AT is composed of different cell types^13^, the consequent cross-talk could hamper a transparent view of human adipocytes. Hence, we chose to study human primary adipocytes alone to avoid AT complexity and obtain a clear detailed picture of human adipocytes and its link with obesity and insulin regulation.

## Results

**Figure 1:**
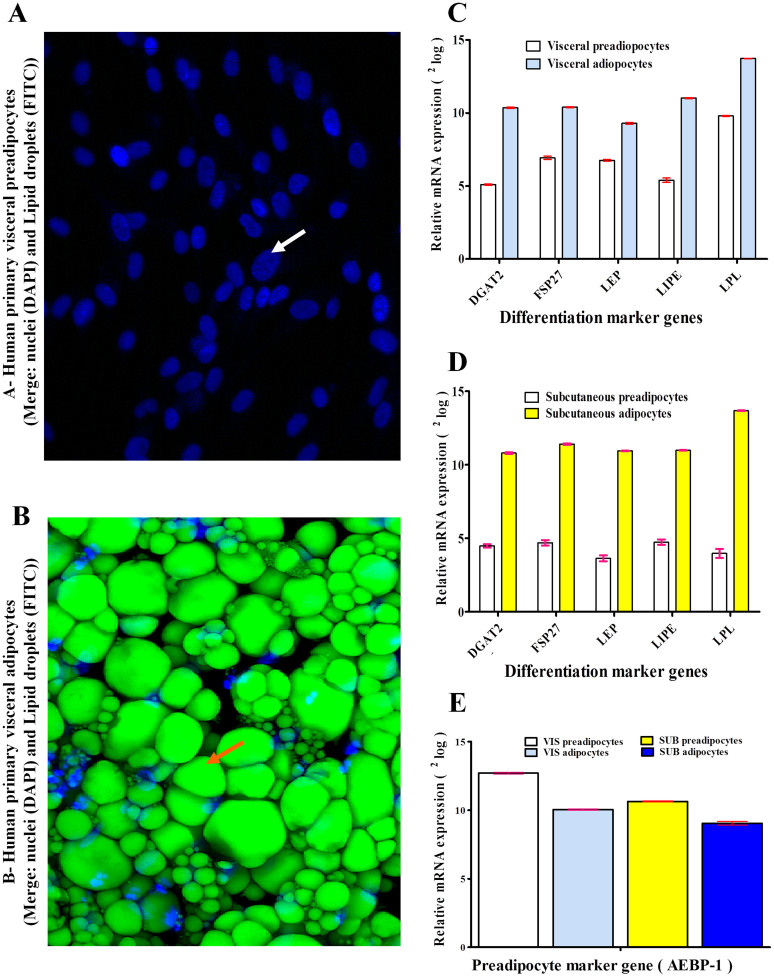
(A–E). Differentiation of human visceral preadipocytes to adipocytes. (Panel A) shows human pre-adipocytes (no lipid droplets) and (Panel B) exhibits adipocytes (with medium-sized or single large lipid droplets). In panel A, nuclei and lipid droplets were stained with DAPI and FITC respectively. Nuclei were indicated by white arrow. In panel B, lipid droplets were stained with FITC and indicated by orange arrow. Almost the whole space of adipocytes is occupied by lipid droplets. Immune fluorescent confocal laser scan microscopy (IFCLSM) was applied to detect DAPI and FITC. (Panel C and D) display 5 differentiation marker genes for human primary visceral and subcutaneous adipocytes respectively**.** mRNA expression was expressed as^2^log (for example: the difference between human subcutaneous preadipocytes and adipocytes regarding leptin (*LEP*) expression is approximately 7, thus true difference is 2^7^ ( = 128-fold) and was shown in y-as. Diacylglycerol O-acyltransferase 2 (*DGAT2*), hormone-sensitive lipase (*LIPE*), lipoprotein lipase (*LPL*) and fat specific protein-27 (*FSP27*). (Panel E) shows marker gene for human primary visceral and subcutaneous preadipocytes. Adipocyte enhancer-binding protein 1 (*AEBP-1*) was higher in human preadipocytes than human adipocytes.

Human primary preadipocytes were differentiated into adipocytes and the pure adipocyte fractions were assessed by monitoring morphologically and via lipid droplet labeling (Figure 1 A–B). The efficacy of differentiation reached approximately 90–95%, indicating that adipocytes could be considered as a specific homogenous cell type. As depicted in Figure 1 A, no lipid droplet was observed and/or detected in human preadipocytes. On the contrary, human adipocytes were occupied by either medium-sized or single large lipid droplets (Figure 1 B).

To confirm our microscopy approach to assess adipocyte-fraction purity, the expression of five known mRNA markers for human adipocytes and one for preadipocytes were measured. mRNA expression displayed a significant up-regulation of the five mRNA markers in both visceral and subcutaneous adipocytes, i.e. *DGAT2*, *LEP*, *LIPE*, *LPL* and *FSP27* (Figure 1 C–D) as compared to human preadipocytes, and *AEBP-1* was significantly up-regulated in both human preadipocytes as compared to human adipocytes (Figure 1 E).

**Figure 2:**
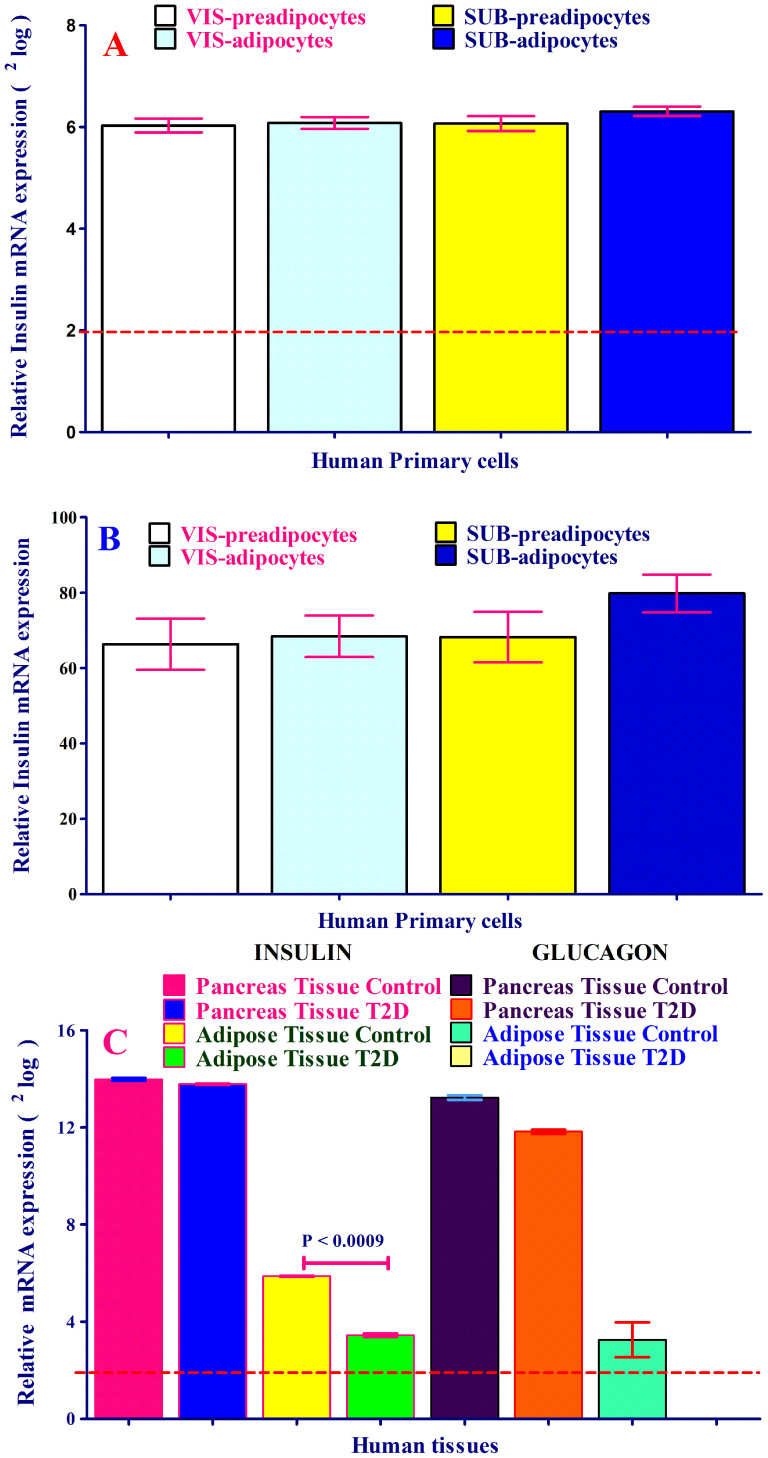
(A–B). mRNA expression analysis of insulin (***INS***) in human primary subcutaneous and visceral preadipocytes and adipocytes. Relative Insulin mRNA levels expressed as^2^log in (Panel A) and as relative intensity in (panel B). mRNA chip (human gene chip) approach was used to detect insulin mRNA expression. Each sample was measured 4 times by mRNA expression assay (human gene chip). (Panel C). mRNA expression of insulin and glucagon in human pancreas and adipose tissue (AT) with and without type 2 diabetes (T2D). The mRNA expression levels of insulin and almost glucagon remained unchanged in pancreas of either state. The mRNA expression of insulin was decreased and glucagon demolished in AT-T2D as compared to AT without T2D.

We investigated whether human primary subcutaneous and visceral (preadipocytes and adipocytes) cells express insulin (*INS*) mRNA and, in turn, translated to insulin protein. All four tested cell types expressed insulin mRNA as determined by mRNA expression-arrays (Figure 2 A–B). As positive control and validation of obtained results by human preadipocytes/adipocytes, we have measured the *INS* (β-cells) and glucagon (*GCG*) (α-cells) mRNAs in human pancreas and AT. The relative intensity of *INS* and *GCG* mRNAs in pancreas tissue reached saturated state. *INS* mRNA expression in human pancreas was approximately 256-fold and 1100-fold higher than *INS* mRNA expression in human AT-control and AT-T2D respectively, but comparable with human pancreas derived from human subject with T2D (Figure 2 C). Importantly, *INS* mRNA expression was reduced in human AT-T2D as compared to the AT-control (Figure 2 C). Of note, *INS* mRNA was expressed in both ATs (Figure 2 C). As shown in figure 2 C, *GCG* expression was completely diminished in AT-T2D as compared to AT-control. *GCG* mRNA expression was also reduced by approximately 3-fold in pancreas with T2D comparing with pancreas control tissue. To determine the specificity of *INS* gene expression, we also measured *IGF1* and *IGF2* expression in pancreas and AT in either state. The expression of these three genes was completely different in pancreas and AT in both control and T2D states (Figure 1, SI).

**Figure 3:**
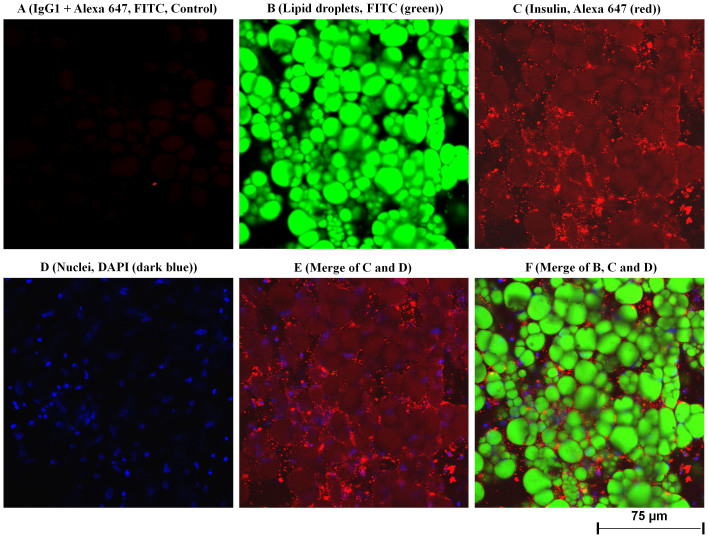
(A–F). Immunofluorescent confocal laser scanning microscopy (IFCLSM) analysis of human visceral adipocytes. From A to F, confocal microscopy analysis was applied to detect and localize insulin protein in the human primary visceral adipocytes. The cultured adipocytes in six-well plates were incubated with IgG1 isotype and the detection antibody Alexa 647 (A; considered as negative control), lipid droplets were visualized with FITC (B; green color); adipocyte was incubated with monoclonal antibody against human insulin (C, red color). Bound antibodies were detected with Alexa 647 coupled goat anti-mouse (C; red color); adipocytes were stained with DAPI to detect nuclei (D, blue color), C (insulin) and D (nuclei) were merged (E), and B (lipid droplets), C (insulin) and D (nuclei) exhibit a merge of all three labeling (F). Insulin protein was located on the surface of plasma membrane and cytoplasm (C, E and F) of human visceral adipocytes. Fluorescent labeling was used for all detections. Original magnification was 400 times. The same results were found for human subcutaneous adipocytes (Figure 2 A–G, SI).

**Figure 4:**
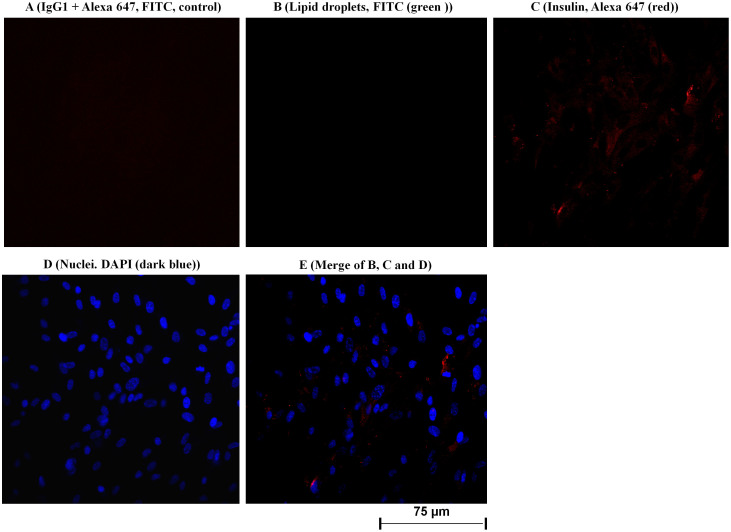
(A–E). Confocal microscopy analysis was applied to detect and localize insulin protein in the human primary visceral preadipocytes. The cultured preadipocytes in six-well plates were incubated with IgG1 isotype and the detection antibody Alexa 647 (A; considered as negative control), lipid droplets were stained with FITC (B; green color); adipocyte was incubated with monoclonal antibody against human insulin. Bound antibodies were detected with Alexa 647 coupled goat anti-mouse (C; red color); preadipocytes were stained with DAPI to detect nuclei (D, blue color). E (merge) exhibits a merge of all three labeling; B (lipid droplets), C (insulin) and D (nuclei). Insulin protein was located in cytoplasm and on the surface of plasma membrane (C and E) of human visceral preadipocytes. Fluorescent labeling was used for all detections. Original magnification used was 400 times. The same results were found for human subcutaneous preadipocytes (Figure 3 A–G, SI).

To determine whether the *INS* mRNA is translated into protein, immunofluorescent confocal laser scanning microscopy (IFCLSM) was applied to human subcutaneous and visceral (preadipocytes and adipocytes) cells. Indeed, IFCLSM analysis revealed the huge insulin protein staining in the cytoplasm and on the plasma membranes of human visceral adipocytes (Figure 3 A–F) and subcutaneous adipocytes (Figure 2 A–G, SI), while insulin protein expression in both human visceral (Figure 4 A–E) and subcutaneous (Figure 3 A–G, SI) preadipocytes were traces. IgG1 isotype controls were all negative (preadipocytes and adipocytes) (Figure 3 A and 4 A). To validate our findings that human adipocytes express insulin, immunoelectron microscopy (IEM) was also applied to the primary visceral adipocytes. As expected, IEM analysis (Figure 4B, SI) showed insulin protein expression in human adipocytes (conjugated with 15 nm gold particles and visualized as black dots) similar to IFCLSM observations (Figure 3 C). IgG1 isotype control was also negative (Figure 4 A, SI).

**Figure 5:**
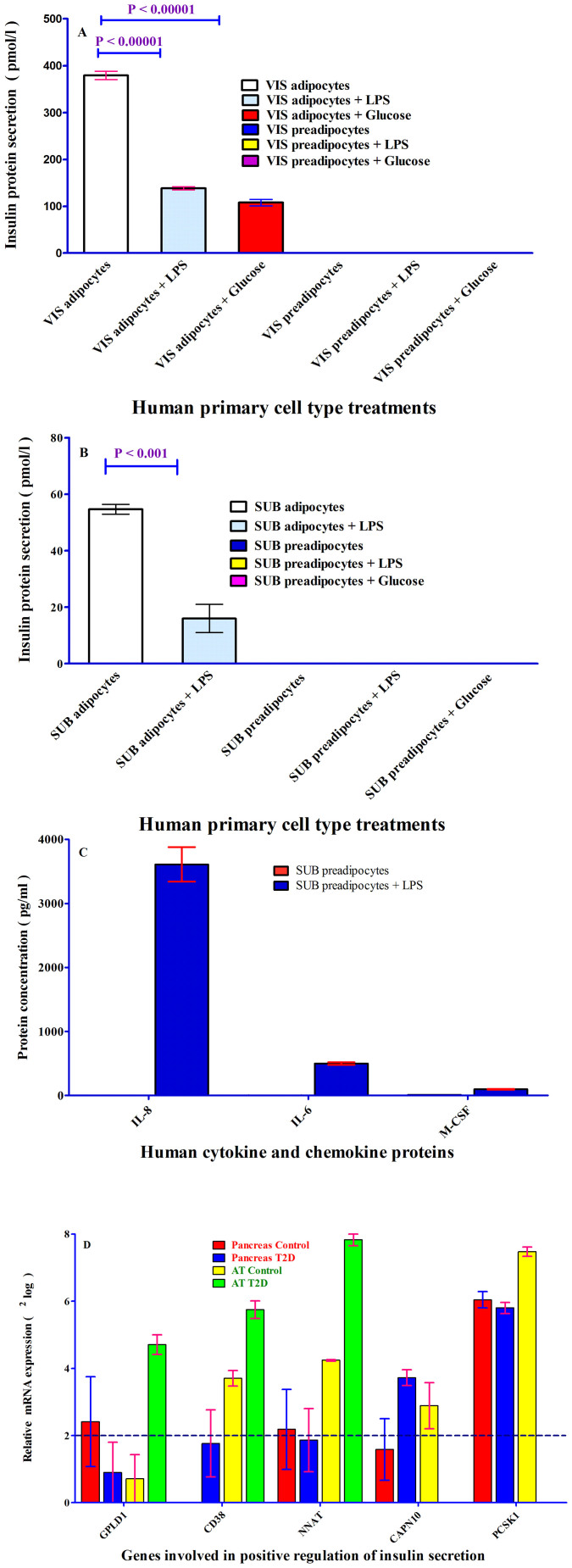
(A–B). ELISA Analysis of insulin in secreted fraction collected from human primary preadipocytes and adipocytes. ELISA analysis of secreted fractions collected from with and without lipopolysaccharides (LPS (200 ng/ml)) and 25 mM glucose treated human primary visceral and subcutaneous preadipocytes and adipocytes. DAKO insulin ELISA kit was applied to measure insulin concentrations in the secreted fractions. Two independent ELISA was used to measure insulin concentrations in secreted fractions; insulin concentration in secreted samples was performed respectively in single and duplicate by ELISA. Insulin concentrations were expressed in pg/ml as shown in Y-axis. (panel A); visceral cells and (Panel B). subcutaneous cells. The p-value obtained from an independent two-tailed test samples (T-Test). P < 0.05 was accepted as statistically significant. (Panel C). IL-6, IL-8 and M-CSF analysis in human primary subcutaneous preadipocytes. IL-6, IL-8 and M-CSF were measured in human primary subcutaneous preadipocytes, using Bio-plex according to instructions provided by Bio-Legend. Red bars represent human preadipocytes without LPS treatment and dark blue bars show human preadipocytes treated with LPS. The concentrations of these three cytokine and chemokines were expressed as pg/ml shown in Y-axis. Samples were measured in duplicate. (Panel D). mRNA analysis of genes involved in positive regulation of insulin secretion, glucose induced insulin stimulation in pancreas, and biosynthesis of insulin. *GPLD1*, *CD38*, *NNAT*, *CAPN10* and *PCSK1* genes were measured in human pancreas and AT with and without T2D. The first four genes are involved in positive regulation of insulin secretion and PCSK1 is involved in biosynthesis of insulin via proinsulin conversion. Glucagon (GCG) was depicted in Figure 2 C. mRNA expression expressed as^2^log. Each sample was measured two or three times by mRNA expression assay for pancreas and AT respectively.

To investigate, whether insulin is secreted from human primary subcutaneous and visceral cells, insulin protein levels were measured in the media containing the secretion from all four cell types, using DAKO Insulin ELISA KIT. As depicted in figure 5 A–B, insulin protein was detected in the media of human primary visceral (379 pmol/l) (Figure 5 A) and subcutaneous (55 pmol/l) (Figure 5 B) adipocytes. Notably, no insulin protein was detected in both human primary preadipocytes (Figure 5 A–B).

To further establish the link between fat mass and insulin secretion levels, we chose to stimulate all four cell types with LPS (200 ng/ml) and 25 mM glucose. Upon LPS treatment, insulin protein levels secreted by human subcutaneous and visceral adipocytes were reduced approximately three-fold (Figure 5 A–B). Treatment of human primary visceral adipocytes with glucose resulted in an approximately four-fold less insulin protein concentrations than the control (untreated with glucose) (Figure 5 A).

To determine, whether the lack of undetectable insulin secretion is a specific feature of human preadipocytes, IL-6, IL-8 and M-CSF protein concentrations were measured in the secretion of only human subcutaneous preadipocytes treated with and without LPS. IL-6, IL-8 and M-CSF proteins were notably detected and measured in the secretion of human subcutaneous preadipocytes without LPS treatment (2.5 pg/ml), (0.0 pg/ml) and (10.5 pg/ml) and treated with LPS (499 pg/ml), (3610 pg/ml) and (97.7 pg/ml) respectively (Figure 5 C).

To gain more knowledge about insulin secretion regulation by human AT as whole, 6 genes (based on the gene ontology (http://www.expasy.org/)) of which 4 genes positively involved in the insulin secretion and two of them *PCSK1* (Proprotein convertase 1 (PC1)) and *GCG* (glucagon) (Figure 2 C) involved in the regulation of insulin synthesis were measured in pancreas and AT with and without T2D. *GPLD1*, *CD38* and *NNAT* mRNAs were not expressed or close to threshold in pancreas tissues with and without T2D, while the same genes were highly expressed in AT-T2D as compared to AT-control (Figure 5 D). On the contrary, *CAPN10* was up-regulated in pancreas T2D and down-regulated in AT-T2D as compared with control tissues (Figure 5 D). *PCSK1* as key enzyme involved in biosynthesis of insulin was diminished in AT-T2D as compared to AT-control. However, *PCSK1* expression remained unchanged in pancreas in either state (Figure 5 D).

**Figure 6:**
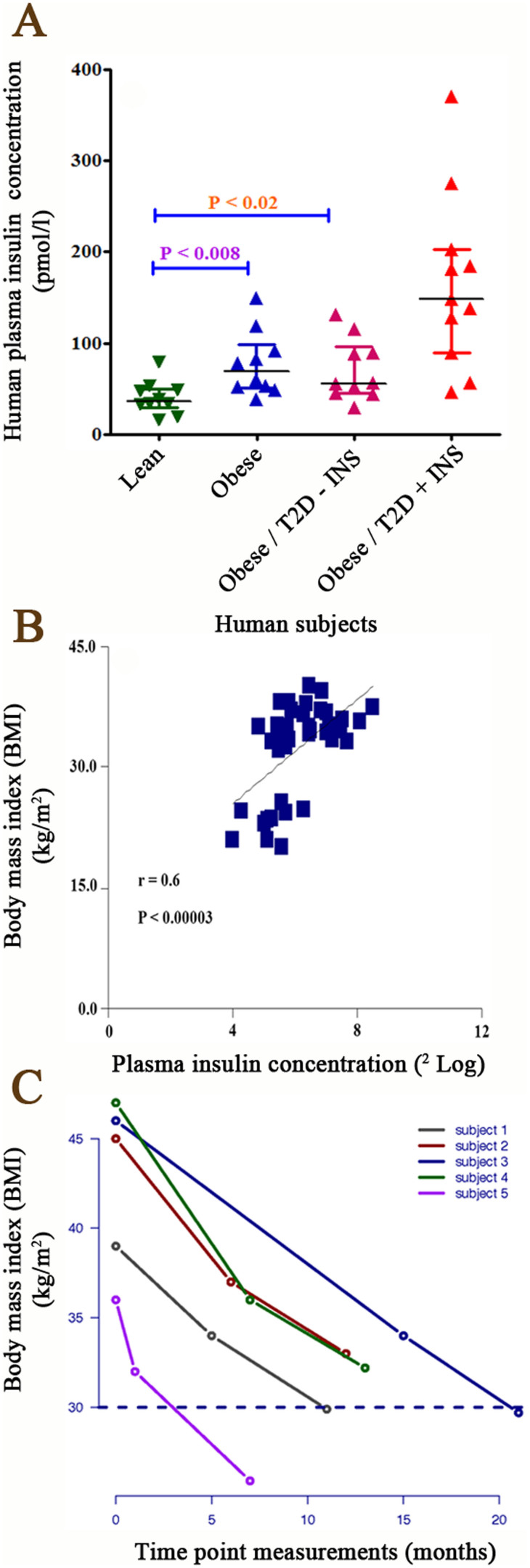
(Panel A). DAKO insulin ELISA kit was applied to measure insulin concentrations in the plasma of 41 human subjects categorized in four groups based on Body Mass Index (BMI); 1- Lean subjects (BMI, 20.1–25.6, only one patient above 25), 2- Obese subjects (BMI, 32.5–39.5), 3-Obese subjects with type 2 diabetes (T2D) without receiving insulin (BMI, 32.1–40) and obese subjects with T2D, receiving insulin (BMI, 33.3–38). BMI is expressed as mass (kg)/(height (m))^2^. The p-value resulted from one-way analysis of variance (ANOVA). P < 0.05 was accepted as statistically significant. Insulin concentrations were expressed as pmol/l shown in Y-axis. (Panel B). Correlation between plasma insulin concentrations (*n* = 41 human subjects) and Body Mass Index (BMI). BMI is expressed as mass (kg)/(height (m))^2^. The p-value resulted from one-way analysis of variance (ANOVA). P < 0.05 was accepted as statistically significant. Insulin concentrations were expressed as pmol/l shown in Y-as and BMI was expressed as^2^log shown in X-axis. There was a strong positive correlation between plasma insulin concentration and human BMI (P < 0.00003, r = 0.6). (Panel C). The bariatric surgery of 5 human subjects with type 2 diabetes. The BMI (fat reduction) was measured three times in 5 patients; 1- before bariatric surgery is considered as basis point, 2- after surgery (depends on the date of surgery) and 3- the last measurement point (6 months after the second measurement). BMI was sharply decreased after the surgery. The BMI was calculated as Kg/m^2^ shown in Y-axis. The time point 0 is considered as before surgery. The X-axis shows the BMI measurements in different time points.

We hypothesized that insulin protein concentrations are positively correlated with body fat mass. To test whether the suggested hypothesis is true, blood insulin concentrations were measured in 41 human subjects. Interestingly, plasma insulin levels were significantly higher in three groups of obese subjects (1-Obese, 2-Obese with T2D and 3-Obese with T2D and insulin injection) than lean subjects (Figure 6 A). Also, plasma insulin concentrations significantly correlated with fat mass (p < 0.00003, r = 0.6, One way ANOVA) (Figure 6 B).

To confirm our hypothesis, bariatric surgery was applied to treat five human obese (BMI above 35) subjects with T2D. After bariatric surgery, the BMI was tremendously reduced in all subjects from 36–47 to 26–33 kg/m^2^ in obese subjects with T2D and rescued these patients from insulin injection (T2D) (Figure 6 C). The BMI of three human subjects were reduced to even below 30 (one subject just above 25; lean value). The BMI of the other two patients were continually reduced by approximately 30%. A weight loss of 40–60 kg was observed in all subjects.

**Figure 7:**
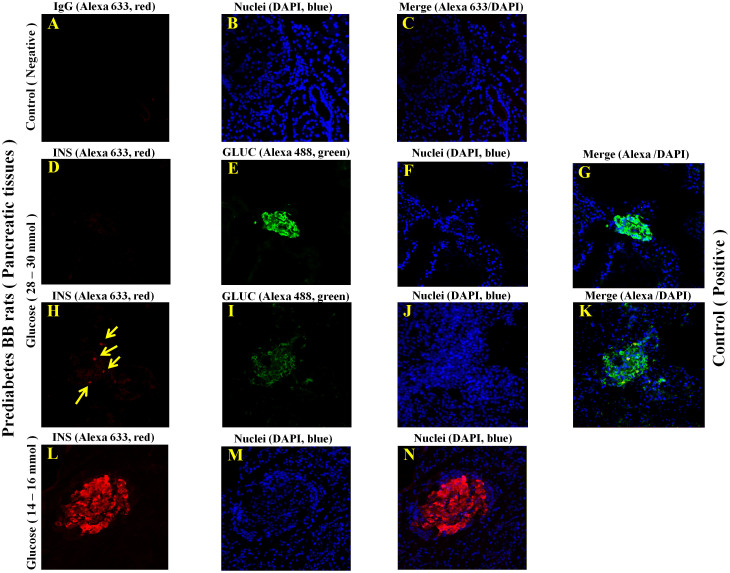
(A–N). The localization and visualization of Insulin and Glucagon in the pancreas tissue cryostats of prediabetes BB rats (T1D-like model) using confocal microscopy strategy. Confocal microscopy analysis was used to localize Insulin and Glucagon in insulin dependent diabetes mellitus (IDDM) and control rats. The glucose concentration of control rats were between 14 and 16 mmol/l. The glucose concentration in IDDM rats were between 28–30 mmol/l. Cryostats were made from pancreas tissues of these rats. The thickness of pancreas cryostats was 5 µm. A cryostat slide was stained only with IgG isotype and alexa 633 coupled goat anti-guinea pig (panel A); considered as a negative control). This slide was not incubated with primary polyclonal antibody. The nuclei were stained with DAPI. Other cryostats were also stained with Polyclonal antibody against rat insulin and bound antibodies were displayed with alexa 633 coupled goat anti-guinea pig (panel D and G; H and K; L and N; red color). Other cryostats were also stained with Polyclonal antibody against rat glucagon and bound antibodies were displayed with alexa 488 coupled goat anti-rabbit (panel E and G; I and K; green color). DAPI was used to stain and detect nuclei in all tissues; F and G; J and L; N and O; dark blue color. Fluorescent labeling was used for all detections. Insulin was not detected in IDDM rats but glucagon (green fluorescent) well. Insulin was found to be positive in islets of Langerhans-associated β cells in control rats. In an islet of Langerhans, four *β* cells were found to be positive for insulin as indicated by 4 light yellow arrows. Glucagon as positive control was found to be positive in the pancreas-associated islets of Langerhans *β* cells in both IDDM and control rats.

To establish the assumption that the production of insulin by human adipocytes is independent of pancreas and provide direct evidence for adipocyte insulin production in a physiological context, we measured *INS* mRNA and insulin protein in pancreas and AT derived from prediabetic BB (Bio-Breeding) rats (type 1 diabetes = T1D-like model). As depicted in figure 7 D and G (in Figure 7 D–G), IFCSLM analysis of rat-derived pancreas tissue cryostats revealed that islets of Langerhans-associated β-cells were destroyed and not detectable in the rats with Insulin Dependent Diabetes Mellitus (IDDM). This finding is in agreement with all other studies, where β-cells were destructed in these rats and not present or detectable. However, islets of Langerhans-associated β-cells were easily detected in control rats by using insulin-specific antibodies and Alexa 633 as detection antibody, as shown in figure 7 L and N. These results were confirmed by showing that glucagon is present and produced by α-cells in islets of Langerhans of rat-derived pancreas tissue as depicted in figure 7 (D-K). We did not observe any signals resulted from IgG (control) or detection antibody (alexa 633) as displayed in figure 7 A. As shown in figure 7 H, four β-cells were detected. This confirmed that almost all islets of Langerhans-associated β-cells were destroyed and also proves that our strategy is specific and works. Although IDDM rats, which do not have β-cells and as a consequence there is no insulin production, AT of both IDDM and control rats produce clearly and convincingly insulin (Figure 5 A and C, D and F, SI), and this insulin production is independent of pancreas-associated islets of Langerhans β-cells. Notably, the staining of insulin in AT of IDDM was much stronger than insulin staining in the AT of control rats (Figure 5, SI).

**Figure 8:**
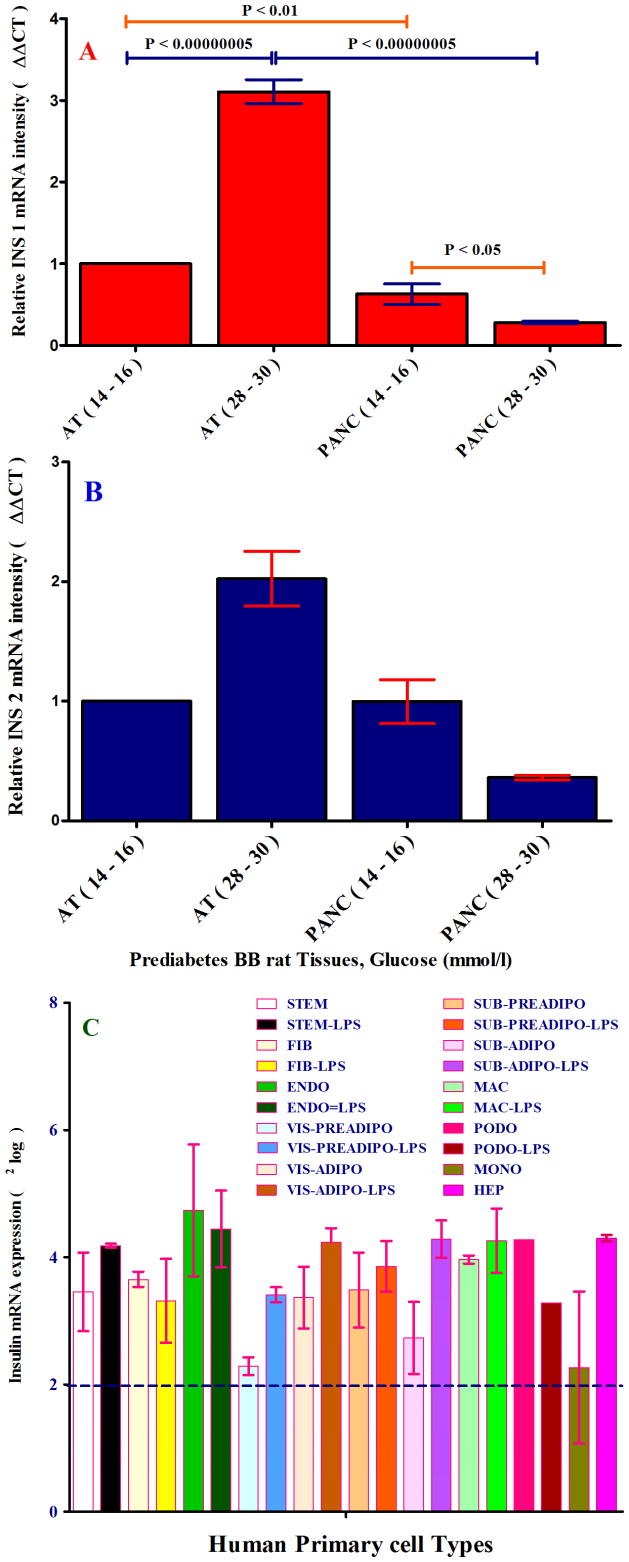
(panel A–B). mRNA expression analysis of insulin1 and insulin2 in prediabetes BB rats (T1D-like). (A), mRNA expression of insulin1 (***Ins1***) and (B), insulin2 (***Ins2***) were measured in pancreas and adipose tissues of IDDM and control rats, using QPCR with Taqman 7900HT. Relative intensity of mRNA was expressed as (ΔΔCt). The amount of the gene of interest (***Ins1 and Ins2*** were normalized to β-actin as endogenous reference and relative to a control sample is 2exp-ΔΔCt. The ΔΔCt is ΔCt “treated” - ΔCt control. AT-control was considered as ΔCt control and tissues with T1D-like were considered as ΔCt treated. After corrections, AT-control was adjusted to 1. Two independent biological replicates were used in mRNA expression analysis. The p-value obtained from an independent two-tailed test samples (T-Test). P < 0.05 was accepted as statistically significant. (Panel C). Expression of insulin by 10 human primary cell types: stem cells, fibroblasts, endothelial cells, macrophages, preadipocytes (Visceral and Subcutaneous; other biological variant) and adipocytes (Visceral and Subcutaneous; other biological variant), hepatocytes, monocytes and one cell line (podocytes). All cells were studied with and without exposure to LPS. mRNA expression of insulin was determined in 11 different human cell types, of which preadipocytes and adipocytes (visceral and subcutaneous) were new biological samples, treated with or without LPS. The level of each mRNA was measured in triplicate (except podocytes) and error bars show standard deviations of means for triplicate cultures per condition and is represented as the mean ± SEM. The p-value obtained from a paired t-test with a two-tailed test samples (T-test). P < 0.05 is considered statistically significant. Insulin mRNA expression is expressed as^2^log values on the Y-axis. Insulin mRNA expression was detected in all cells tested (except traces for monocytes). Relative mRNA intensity was performed triplicat.

To confirm the data obtained by IFCLSM analysis of pancreas cryostats and AT, the total RNAs were collected from the same tissues derived from the same rats for mRNA analysis (QPCR). Since the rats have two insulin genes, ***Ins1*** (P01325) and ***Ins2*** (P01326), both ***Ins1*** and ***Ins2*** mRNAs were also measured in pancreas and AT. The relative intensity of mRNA Ins1 and Ins2 were the highest in the AT and lowest in pancreas tissues of IDDM rats as depicted in figure 8 A-B. Surprisingly and unexpectedly in IDDM rats, which have no or very low number of β-cells, the mRNA expression of *Ins1* is very high in the AT of IDDM rats (Figure 8 A), whereas control rats, which appeared to have enough β-cells, the mRNA expression of ***Ins1*** is low in the AT of control rats. We did perform this experiment for *Ins2* too (Figure 8 B).

Since human adipocytes are a source of insulin production, we sought to investigate if other human primary cell types express insulin. Thus, we examined 6 other human primary cell types (plus 4 subcutaneous and visceral (preadipocytes and adipocytes) from two new biological variant) as well as one cell line (Podocytes) for insulin production (Figure 8 C). Notably, as depicted in figure 8 C, insulin was expressed by almost all cells used in this study. Although the *INS* gene expression is very high in pancreas β-cells and it is not comparable with the expression of insulin by other cell types and AT, the other cells are able to express insulin.

To check whether *INS* gene expression is biologically active, all cells were treated with LPS. Upon the LPS treatment, *INS* mRNA expression increased or decreased as compared to the cells without LPS treatment such as visceral preadipocytes and visceral preadipocytes plus LPS (Figure 8 C).

## Discussion

This study was designed to unravel the role of adipocytes behind the link between obesity and insulin regulation in human. The purity and specificity of preadipocytes and adipocytes fractions were established using two independent techniques: IFCLSM and 6 preadipocytes/adipocytes gene markers. The mRNA expression data convincingly showed the expression of *INS* gene in all 16 measurements (preadipocytes and adipocytes of both subcutaneous and visceral cells, first experiment). Notably, an intense insulin protein staining was present in cytoplasm and plasma membrane of human visceral and subcutaneous adipocytes, but not in human preadipocytes, and that is due to the presence of large lipid droplets (fat storage depot) within adipocytes. Also, these data were confirmed by IEM and IFCSLM. Last but not least, we observed the secretion of insulin by both human adipocytes (subcutaneous and visceral) and no insulin secretion by both human preadipocytes (subcutaneous and visceral). Hence, it seems reasonable to conclude that we could not detect insulin in human preadipocytes-secreted fractions because of the absence of lipid droplets (energy storage depot). Although Gerozissis^14^ and Kojima *et al.*^15^ suggested the extrapancreatic insulin production; our data confirmed not only the concept suggested by Kojima *et al.* but also the insulin production by adipocytes and other cells in normal state and mechanism behind that. Although insulin is similar to insulin-growth factor 1 and 2 (IGF-1 and IGF2) in sequence and structure, which lays out the ground of cross-reactivity of *INS* mRNA with that of *IGF1* and *IGF2*, the selected probe sequence for *IGF1* and *IGF2* genes was different from insulin probe sequence (only approximately 25–28% sequence homology between these two probes and Insulin). This indicates that insulin probe is specific for insulin gene and do not react with *IGF1* and *IGF2*. Also the pattern expression of these three genes are completely different in pancreas and AT in either state, confirming that there is no cross-reactivity between these three mRNAs.

Insulin secretion was reduced upon treatment of human adipocytes with LPS or glucose. Based on published data, both LPS and glucose^16^,^17^,^18^,^19^ induce inflammation, which in turn results in an increased lipolysis^20^,^21^,^22^. As a consequence, fat mass homeostasis would be disrupted and in line with our initial observations, no insulin secretion by human preadipocytes (lacking lipid droplets) would occur. Consistently, the results obtained by visceral adipocytes treated with glucose showed almost the same pattern as β-cells treated with glucose *in vitro*^23^. Since the stimulation of cells with glucose results in an inflammatory state similar to the effect of LPS, and this similarity extends to insulin levels, and given that IL-6 and IL-8^16^ were clearly and convincingly expressed by human preadipocytes, the absence of insulin secretion by human preadipocytes is a specific phenomenon and therefore human preadipocytes can be considered as a good negative cell control. The reason why we have chosen to measure IL-6 and IL-8^16^ is that these cytokines have already been established to be secreted by human preadipocytes. Notably, since other cells express insulin except monocytes, the other cells can be considered as positive control. Since monocytes are circulating cells, it is reasonable to assume that monocytes do not express insulin.

We found that human primary adipocytes constitutively produce and release very low but not trivial amounts of insulin. This intriguing discovery helps us to reshape thinking that explains how obesity could increase the risk for developing T2D.

Since blood insulin levels were high in all human obese subjects as compared to lean subjects and showed a highly significant positive correlation, our suggested hypothesis that blood insulin concentrations increase with the increase of body fat mass is indeed true. It is then possible to suggest that insulin production by obese AT increases the risk for T2D. This positive correlation appears to be in apparent agreement with our *in ex-vivo* observations where we were unable to detect insulin protein by human primary preadipocytes (no lipid droplets). Finally, it must be stressed that our hypothesis regarding insulin and lipid droplets was also confirmed by *in vivo* studies by Puri *et al*.^24^,^25^,^26^,^27^. These authors have shown that FSP27 protein promotes energy reservoir in the form of TGs within lipid droplets and knock out of *Fsp27* gene in mice led to the increase of lipolysis, protecting mice from diet-induced obesity and IR. Since i) the expression of three genes (*GPLD1*, *CD38* and *NNAT*) with a vital role in the positive regulation of insulin secretion are absent in pancreas, but highly up-regulated in AT-T2D, ii) the expression of *CAPN10* gene (with same function as other three genes) was highly up-regulated in pancreas-T2D, but completely diminished in AT-T2D, iii) the expression of *PCSK1* (vital enzyme in proinsulin conversion) is highly expressed in pancreas in either state, but demolished in AT-T2D, and iv) the same pattern was observed for *GCG*, it is reasonable to postulate that pancreas and AT “talk” with each other to ensure a homeostatic energy metabolic system in normal state or minimize the damage in a diabetic state.

A reduction of fat mass was found in 5 patients with T2D after bariatric surgery. Intriguingly, the fat loss rescued and repaired these human subjects from T2D, ensuring “a great feel of freedom and relief”. These data indicate that fat loss in these subjects potentially resulted in a reduction of insulin production by AT, which in turn restore the insulin ability of pancreas-associated β-cells to adjust blood glucose without external insulin injection, confirming our above suggested hypothesis that high concentration of insulin by obese AT deteriorates the insulin function by pancreas β cells. This finding suggests a direct physiological role of insulin produced by AT. However, this hypothesis remained to be in-depth elucidated.

Since *INS* mRNA and protein expression were tremendously higher in AT-T1D-like rats than control rats, these results provide a direct evidence for physiological role of AT-insulin production. Also, the *INS* mRNA expression was decreased in AT-T2D as compared to control subject, while the *INS* mRNA expression in human pancreas (saturated state) remained equal between control and pancreas-T2D.

Of note, the expression of insulin was observed in different human primary cell types and that makes us to re-design our investigations related to insulin and its linkage with diabetes in future studies.

The results presented in this study indicate that the constitutive synthesis of insulin by adipocytes and other cells apparently smoothen the dynamic regulation of insulin levels by the β-cells of the endocrine pancreas and thus provide a plausible mechanism as to how obesity (fat mass) could potentially cause insulin resistance. AT may be also considered as compensate endocrine organ for energy metabolic disorders. Lipid droplets show a link with the regulation of insulin synthesis in human adipocytes.

This study provides a novel knowledge about the role of adipocytes in human insulin regulation and opens a new avenue towards the therapy of T2D in the future.

## Methods

Four different models were used. The description of methods is given in this section.

### *In vivo* (five different groups of human subjects)

41 human subjects divided into four groups of 10 (except diabetes group receiving insulin containing 11 subjects), independent of age and gender, as follows: Group 1-Lean subjects with a body mass index (BMI) between 20.1 and 25.6, Group 2-Obese subjects with a BMI above 30, Group 3- Obese subjects with T2D without receiving insulin and a BMI above 30 and Group 4- Obese subjects with T2D, receiving insulin and BMI above 30. All patients were well-matched for their BMI. It must also be stressed that in lean group only one person has a BMI above 25 (BMI = 25.6). A fifth group of human subjects includes five human subjects with T2D and a BMI of over 35 but independent of age and gender were used to measure blood insulin and BMI after obesity surgery.

### *Ex Vivo* (Human Primary cell types)

10 human primary cell types^28^, one human immortalized cell line (Podocytes) was kindly provided by professor M.A. saleem^29^, were used in this study. These human primary cells are: 1-Poietics™ visceral preadipocytes (LONZA, USA/Belgium), 2-adipocytes (preadipocytes differentiated to adipocytes), 3-subcutaneous preadipocytes (LONZA, USA/Belgium), 4-subcutaneous adipocytes (preadipocytes differentiated to adipocytes) 5-bone marrow derived human primary mesenchymal stem cells (hMSC-BM) (PromoCell, Germany), 6-dermal fibroblasts (PromoCell, Germany), 7-umbilical vein endothelial cells (HUVECs) (University Medical Center Groningen (UMCG) endothelial Facility), 8-peripheral blood CD14+ monocytes (LONZA, USA/Belgium), 9-macrophages (monocytes differentiated to macrophages by phorbol-12-myristate-13-acetate (PMA), and 10-hepatocytes (Becton & Dickinson (BD) USA/Germany). Human primary preadipocytes were differentiated into adipocytes as described by Meijer *et al*.^30^ and the instructions of PromoCell Company. To this end (first experiments), stringent criteria were applied to prevent or suppress the false positive and artifact results in the human primary subcutaneous and visceral (preadipocytes and adipocytes). Human primary subcutaneous and visceral (preadipocytes and adipocytes) RNAs were isolated from two independent technical replicates and each replicate was performed in duplicate and each sample was measured in human chip in duplicate. Finally, each cell type was measured four times (in total 16 times). Also, the gene expression was expressed as^2^log to suppress false positive results derived from intrachip comparison as well as the up-regulation and down-regulation of genes are treated in a similar fashion^31^.

### Preadipocytes and adipocytes markers

Five mRNA markers of both visceral and subcutaneous adipocytes, (i.e. Diacylglycerol O-acyltransferase 2 (*DGAT2*; accession number Q96PD7), leptin (*LEP*; P41159), hormone-sensitive lipase (*LIPE*; Q05469), lipoprotein lipase (*LPL*; P06858) and fat specific protein-27 (*FSP27*; Q96AQ7)^24^,^25^,^26^,^27^,^32^,^33^ (Figure 1 C–D) and one preadipocytes marker; adipocyte enhancer-binding protein 1 (*AEBP-1*; Q8IUX7) were measured^34^.

### Genes involved in positive regulation of insulin secretion, glucose induced insulin stimulation in pancreas, and biosynthesis of insulin

The expression of Six genes were measured in human pancreas and AT with and without T2D. *GPLD1* (P80108)^35^, ADP-ribosyl cyclase 1 (*CD38*; P28907)^36^, Neurontin (*NNAT*; Q16517)^37^, and Calcium-activated neutral proteinase 10 (*CAPN10*; Q9HC96)^38^ were involved in positive regulation of insulin secretion. Glucagon (*GCG*; P01275)^39^ was involved in glucose induced insulin stimulation. Proprotein convertase 1 (*PSCK1*; P29120)^40^ was considered as key enzyme in biosynthesis of insulin via conversion of proinsulin. Last but not least, Insulin (*INS*; P01308) itself was measured. The selection of these genes was based on gene ontology and WWW.expasy.org was applied (Swiss proteomics).

### *In Vivo* (Human pancreas and Adipose tissues)

Human pancreas tissues (control and T2D), and AT (control and T2D) were purchased from AMS Biotechnology (AmsBio, England). *INS* mRNA expression was measured in these tissues. Probe sequence for human *INS* gene is: ACCCGCCGCCTCCTGCACCGAGAGAGATGGAATAAAGCCCTTGAACCAGC and for human *IGF1* (insulin-like growth factor 1) gene is: GAGGCCCAGGGGATTTTTGAAGCTGTCTTTATTCTGCCCCCATCCCAACC. and for human *IGF2* (insulin-like growth factor 2) gene is: AGGGAGGCCAAACGTCACCGTCCCCTGATTGCTCTACCCACCCAAGACCC.

All experiments were performed according to the manual instructions provided by the companies. These three probes were used for all screening of Insulin, *IGF1*, and *IGF2* in pancreas, AT, and preadipocytes and adipocytes and all other cell types used in this study.

The specific monoclonal antibody against human insulin combined with Transmission Electron Microscopy (TEM) and/or confocal microscopy were used to visualize insulin protein in human adipocye slides.

### *In Vivo* (prediabetes BB rat pancreas and Adipose tissues)

Prediabetes BB rats were chosen as model in study because we must have a model that *islets of Langerhans-* associated β-cells are selectively destroyed or not functional T1D-like model). Based on search machine, prediabetic BB (Bio-Breeding) rats are considered as a good and established model for studying insulin-producing β-cell destruction located in the *islets of Langerhans* in pancreas during the development of Insulin Dependent Diabetes Mellitus (IDDM)^41^,^42^,^43^. For this reason, we have chosen for prediabetes BB rats. Total RNAs were collected from prediabetes BB rats (IDDM and control rats were kindly provided by Dr. Visser (“Animal care and handling was in agreement with NIH protocol for animal treatment (NIH publication no. 85–23; revised 1985), and animal experiments were approved by UMCG Ethical Board for Animal Studies”)^44^,^45^. The methods were carried out in "accordance" with the approved guidelines. The glucose concentration was used to monitor rats with IDDM (glucose concentration 28–30 mmol/l) and control (glucose concentration 14–16 mmol/l) rats. Total RNAs were collected from pancreas and adipose tissues to make cDNAs for QPCR. The KAPA SYBR® FAST qPCR Kit (Sopachem) was used for QPCR. Total volume for QPCR was 20 μl. Since the rodents have two insulin genes (*Ins1* and *Ins2*), the relative intensity of both *Ins1* and *Ins2* were measured. Insulin mRNA levels are expressed relative to β-actin mRNA levels. All primers^46^ were HPLC purified. Primer sequences are as follows:

Rat *Ins1* and *Ins2* forward primers: GCCCAGGCTTTTGTCAAACA

Rat *Ins1* reverse primer: GTTCCCCACACACCAGGTAGAGAG

Rat *Ins2* reverse primer: CTCCCCACACACCAGGTAGAG

Rat *β-actin* forward primer: ACGAGGCCCAGAGCAAGA

Rat *β-actin* reverse primer: TTGGTTACAATGCCGTGTTCA

### Experiments

Total RNAs were isolated from human primary cells (Human genome Chip), human ((Human genome Chip) and prediabetes BB rats tissues to use in mRNA expression analysis, applying Nucleospin kit. All experiments were executed according to the instructions provided by Nucleospin. The specific monoclonal antibody against human insulin, rat insulin, and rat glucagon combined with Transmission Electron Microscopy (TEM) and/or confocal microscopy or Immunoelectron microscopy were used to visualize insulin protein in human adipocyes slides. Multi-plex ELISA of IL-6, IL-8 and M-CSF proteins were purchased from Biolegend and the experiments were performed according to the Biolegend instructions.

Before the collection of media for secretion analysis, all media with supplement were withdrawn and the cells were washed 5 times with the differentiation media (without supplement). After wash steps, the cells were incubated with the differentiation medium (without supplement or any other additional components such as antibiotic and phenol red) for 2 days (48 h) and medium collected containing secretion proteins of human preadipocytes/adipocytes. The final media, which was used for incubation of cells for all experiments were blank (contains no supplements, insulin, phenol red, and antibiotica).

The collected media were subsequently concentrated, using a cut-off filter of 3 kDa. This collected media were used for further experiments. The remained cells were also used for imaging.

DAKO insulin ELISA kit was applied to measure insulin concentrations in the secreted fractions.

According to DAKO insulin ELISA kit information and instruction, cross reactivity with “C-peptide at a concentration of 5000 pmol/L was below the detection limit of the insulin assay”. The Dako Insulin assay measures biologically active insulin with a high degree of specificity. All human primary cells were treated with 200 ng/ml lipopolysaccharides (LPS). All collected human preadipocytes- and adipocytes-secreted fractions were already treated with 200 ng/ml LPS or 25 mM glucose, for all human mRNA analyses.

### Ethics statement

This study was approved by the Medical Ethical Committee of the Erasmus Medical Center, Medical University of Rotterdam, under **MEC** number: 2009-242. Written informed consent was obtained from all participants. The methods were carried out in “accordance” with the approved guidelines.

## Supplementary Material

Supplementary Information Supplementary information
