# Supplementary material for: Lipid droplets hypertrophy: a crucial determining factor in insulin regulation by adipocytes
Source: Sci Rep. 2015 Mar 6;5:8816. doi: 10.1038/srep08816 (PMC4649717; doi:10.1038/srep08816)
Supplement: Supplementary Information [file srep08816-s1.pdf]

LIPID DROPLETS HYPERTROPHY: A CRUCIAL DETERMINING FACTOR IN INSULIN REGULATION BY ADIPOCYTES

[1]Bahram Sanjabi[#], [2,3]Monireh Dashty[#], [4]Behiye. Özcan,[5]Vishtaseb Akbarkhanzadeh, [3]Mehran Rahimi, [7,8]Manlio Vinciguerra, [9]Felix van Rooij, [10]Saad Al-Lahham, [10,11]Fareeba Sheedfar, [12]Theo. G. van Kooten, [13]C. Arnold. Spek, [14]Ajda T. Rowshani, [15]Johannes van der Want, [16]Rene. Klaassen, [9]Eric Sijbrands, [2]Maikel. Peppelenbosch, [2,3]Farhad. Rezaee[*]

***Corresponding author**:

Dr. F. Rezaee

Department of Cell Biology, Department of Medical Proteomics, University Medical Center Groningen, University of Groningen, Antonius Deusingslaan 1, 9713 AV, Groningen, The Netherlands. Email: F.rezaee@erasmusmc.nl , F.Rezaee@med.umcg.nl

SI Figures 1-6.

Figure 1. Comparison of mRNA expression between *INS, IGF1 and IGF2* in human pancreas and AT with and without T2D.

Relative *IGF-1*, *IGF-2* and *INS* mRNA levels expressed as ^{2}log. Each mRNA was measured in triplicate.

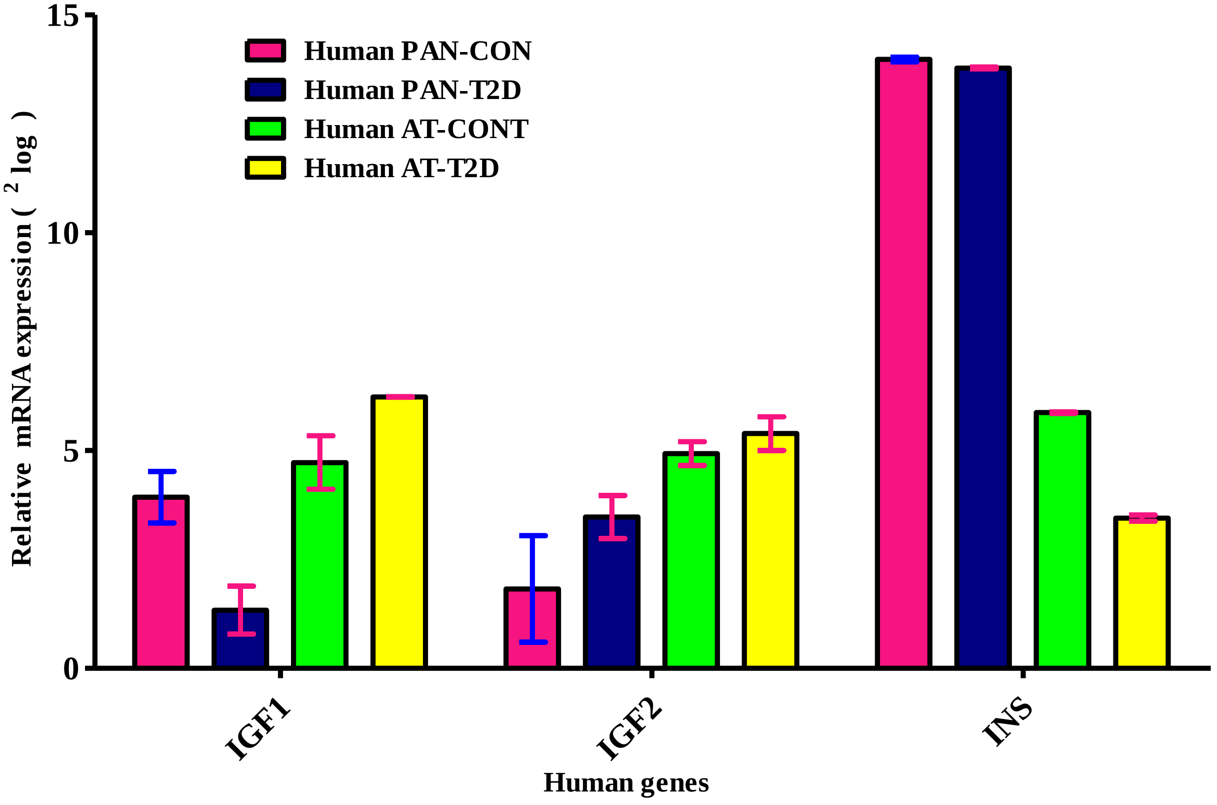

Figure SI-2 A-G. Human primary subcutaneous adipocytes

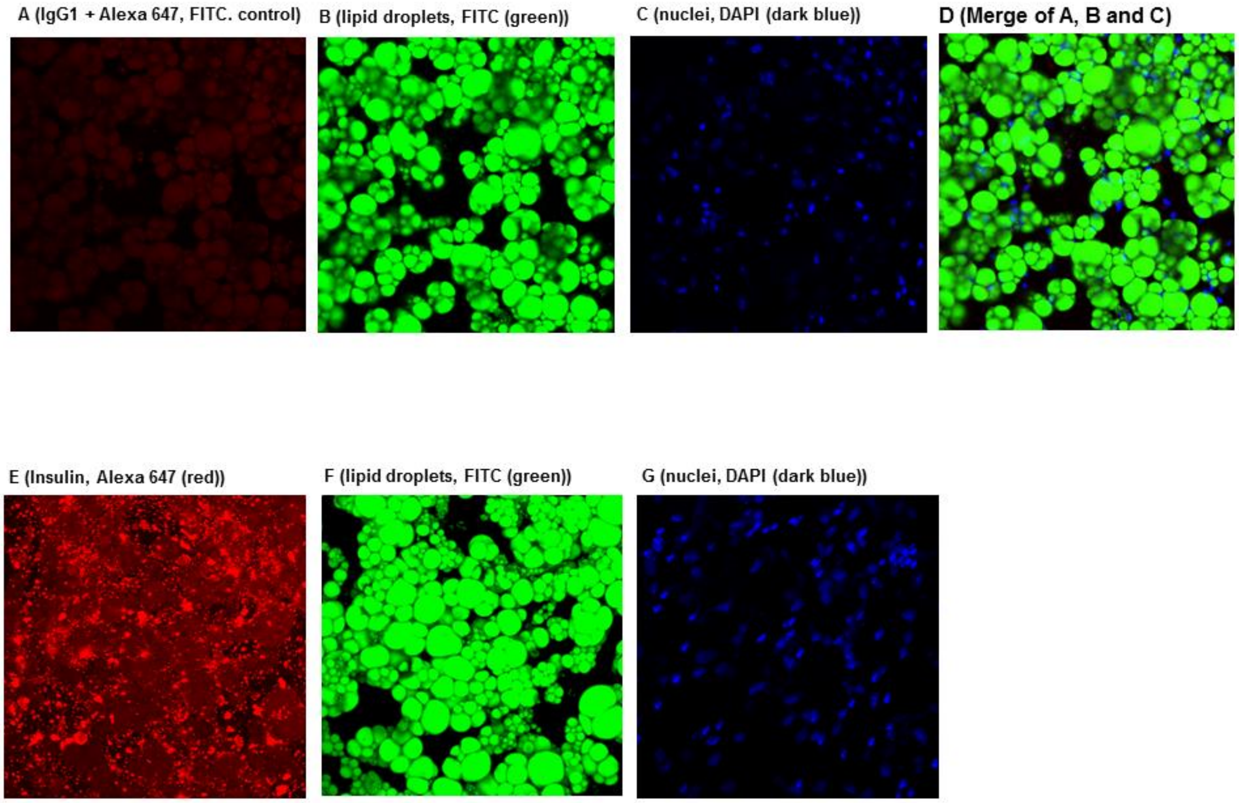

Figure SI-2 A-G. Immunofluorescent confocal laser scanning microscopy (IFCLSM) analysis of human subcutaneous adipocytes. Confocal microscopy analysis was applied to detect and localize insulin protein in the human primary subcutaneous adipocytes. From **A to D (Negative control experiment)**, the cultured adipocytes in six-well plates were incubated with IgG1 isotype and the detection antibody Alexa 647 (**A;** considered as negative control), lipid droplets were visualized with FITC (**B;** green color); adipocytes were stained with DAPI to detect nuclei (**C**, blue color). **A**, **B** and **C** were merged in **D**. Adipocytes were incubated with monoclonal antibody against human insulin (**E, red color).** Bound antibodies were detected with Alexa 647 coupled goat anti-mouse (**E**; red color). Adipocytes were stained with FITC to show Lipid droplet (**F**, green), and DAPI to detect nuclei (**G**, blue color). Fluorescent labeling was used for all detections. Original magnification was 400 times.

Figure SI- 3 A-G. Human primary subcutaneous preadipocytes

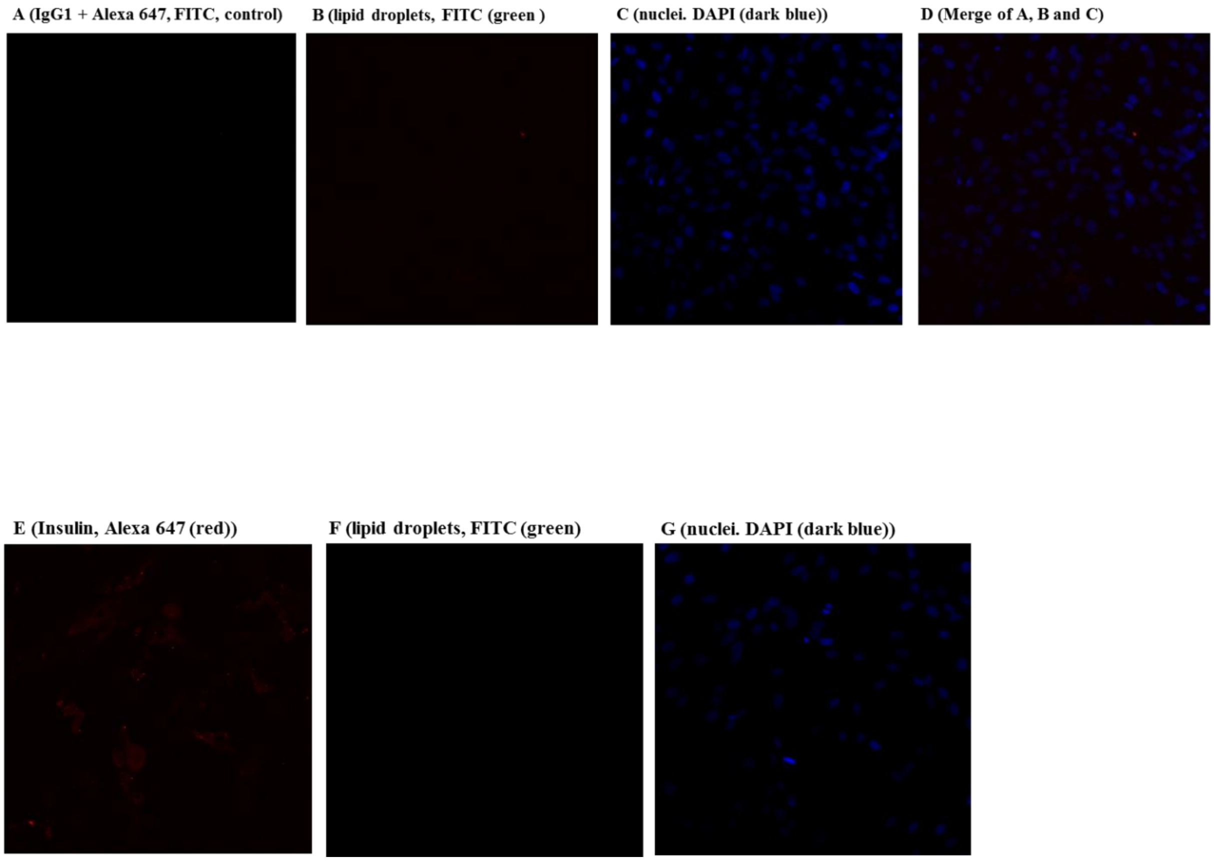

Figure A-G. Confocal microscopy analysis was applied to detect and localize insulin protein in the human primary subcutaneous preadipocytes. The cultured preadipocytes in six-well plates were incubated with IgG1 isotype and the detection antibody Alexa 647 (**A;** considered as negative control), lipid droplets were stained with FITC (**B;** green color) and DAPI to detect nuclei (**C**, blue color). **A**, **B** and **C** were merged in **D**. Adipocytes were incubated with monoclonal antibody against human insulin. Bound antibodies were detected with Alexa 647 coupled goat anti-mouse (**E**; red color); preadipocytes were stained with FITC to visualize lipid droplets (F, green) and DAPI to detect nuclei (**G**, blue color). Fluorescent labeling was used for all detections. Original magnification used was 400 times.

Figure SI-4 A-B. Immunoelectron microscopy analysis of human visceral adipocytes.

Panel A exhibits labelling without specific primary monoclonal antibody against human insulin, but with IgG isotype 1 (considered as control). **Panel B**: labelling with specific primary monoclonal antibody again human insulin detected with gold-conjugated goat anti-mouse. Black dots represent insulin bound gold particles (15 nm).

A- Control (Magnification 45000 X)

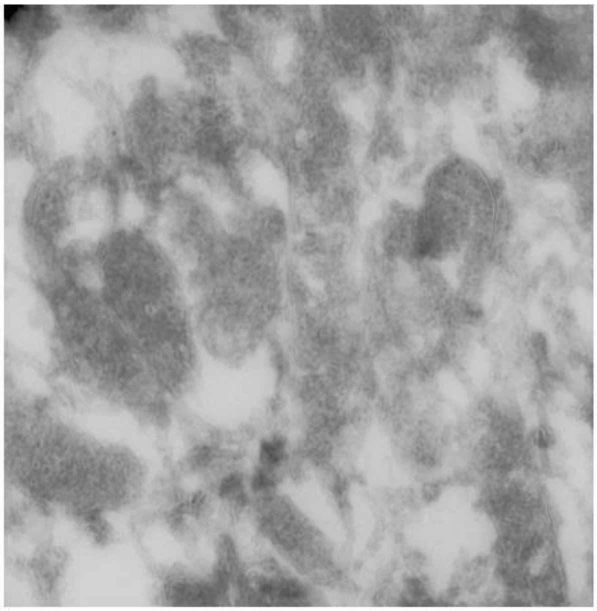

B- Insulin (Magnification 45000 X)

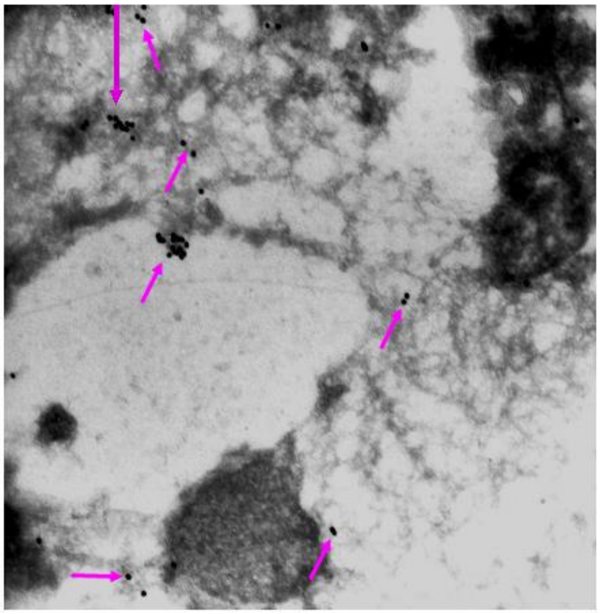

Figure SI-5 A-F. The localization and visualization of Insulin in the fat tissue cryostats of prediabetes BB rats (type 1 diabetes-like = T1D-like) using confocal microscopy strategy.

Confocal microscopy analysis was used to localize Insulin in insulin dependent diabetes mellitus (IDDM) and control rats. Cryostats were made from fat tissues of these rats. The thickness of fat cryostats were 15 µm. Fat cryostats were also stained with Polyclonal antibody against rat insulin and bound antibodies were displayed with alexa 633 coupled goat anti-guinea pig (**panel A**, **C**, **D** and **F**). DAPI was used to stain nuclei in all slides. Insulin was found to be positive in both IDDM and control rats.

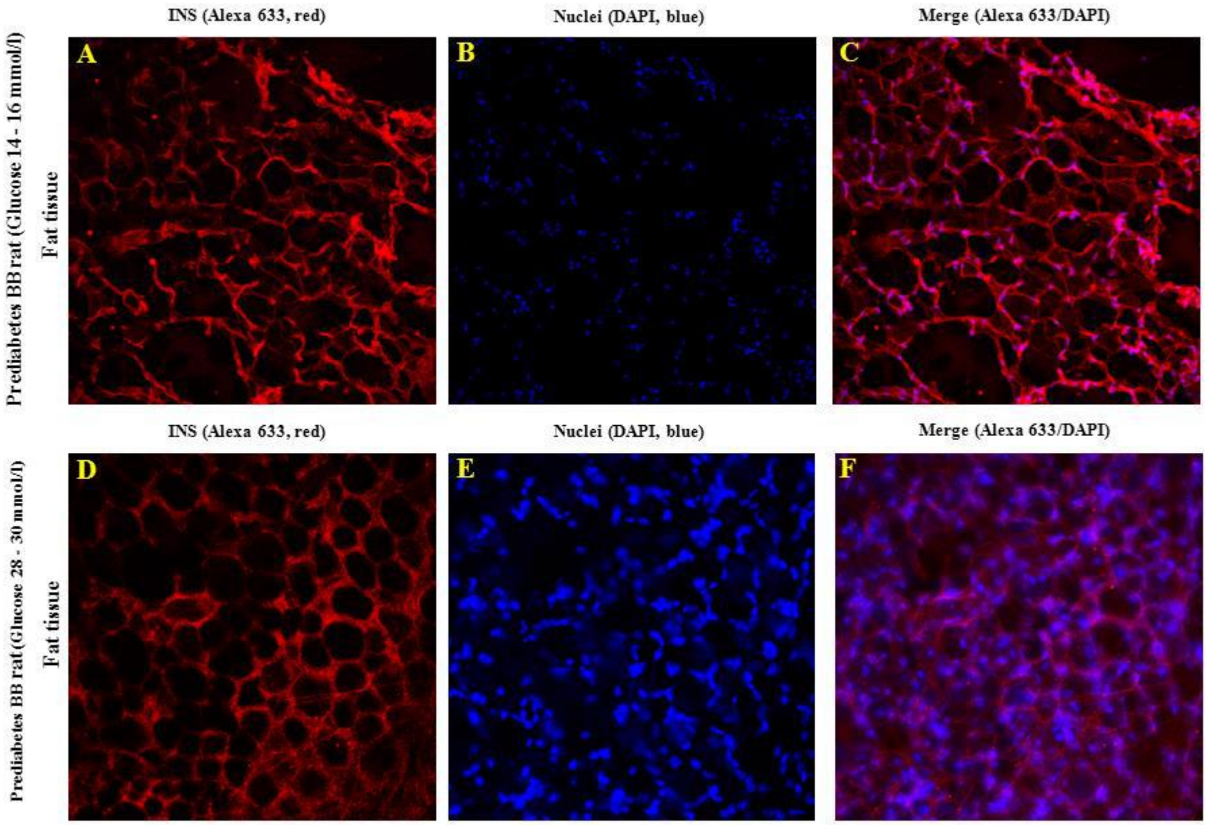

Figure SI-6. mRNA levels of *PPARγ* and the *C/EBPs* in human primary visceral and subcutaneous preadipocytes and adipocytes.

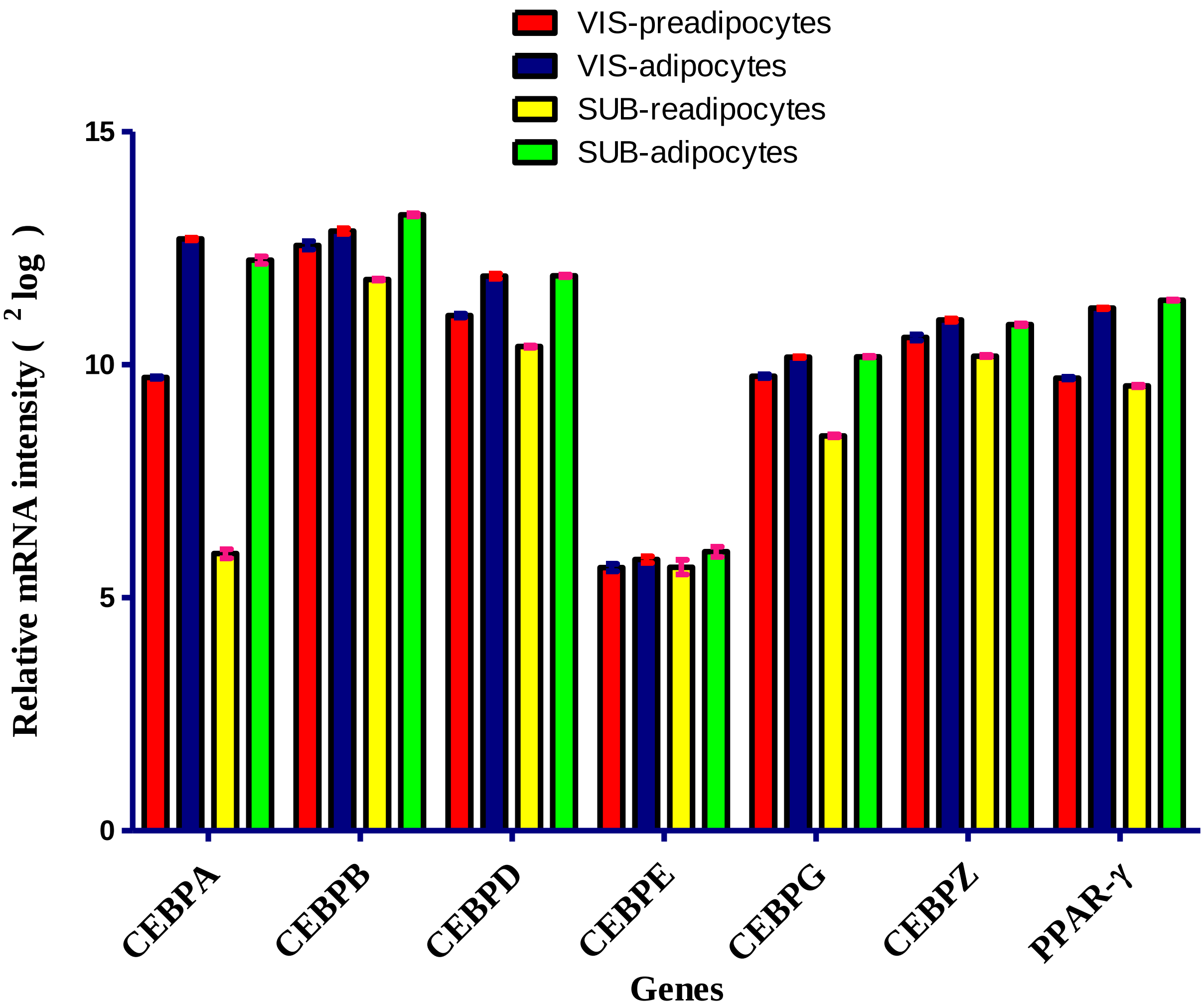

Figure 6. Relative *C/EBPs* and *PPAR-γ* mRNA levels expressed as ^{2}log. Each mRNA was measured in quadruplicate.
